# Exogenous copper oxide nanoparticle treatment (seed and foliar) modifies photosynthetic rate and antioxidant enzyme activity in *Brassica juncea*

**DOI:** 10.3389/fpls.2026.1871020

**Published:** 2026-08-19

**Authors:** Ahmad Faraz, Parisa Ebrahimbabaie, Ferhat Ozturk, Randa A. Zarban, Wael F. Shehata, Jameel M. Al-Khayri

**Affiliations:** 1 Plant Physiology Lab, Department of Botany, Aligarh Muslim University, Aligarh, UP, India; 2 Department of Botany, Shibli National College, U.P., Azamgarh, India; 3 Department of Environmental Science, Bethune-Cookman University, Daytona Beach, FL, United States; 4 Department of Field Crops, Faculty of Agriculture, University of Sirnak, Sirnak, Türkiye; 5 Department of Agricultural Biotechnology, College of Agriculture and Food Sciences, King Faisal University, Al-Ahsa, Saudi Arabia

**Keywords:** antioxidant, nano-enabled agriculture, nanoparticles, photosynthetic enhancement, reactive oxygen species, copper oxide nanoparticles, *Brassica juncea*, seed priming

## Abstract

Nanotechnology facilitates efficient micronutrient delivery to crops, with copper oxide nanoparticles (CuO NPs) identified as promising nanofertilizers. This study investigated the effects of CuO NPs applied via seed soaking and foliar spray on the growth and physiological responses of *Brassica juncea* (mustard). Seeds were soaked in a 4 mg L^−1^ CuO NP suspension for 30 minutes prior to sowing, followed by foliar application of varying CuO NP concentrations at 25 days after sowing (DAS). Growth, photosynthetic, enzymatic, and biochemical parameters were evaluated to identify the most effective application strategy. The combination of 4 mg L^−1^ seed soaking and 8 mg L^−1^ foliar spray yielded the greatest improvements. At 45 DAS, shoot and root lengths, fresh and dry masses, leaf area, and SPAD values increased by approximately 48–51% compared to the control. Photosynthetic rate, stomatal conductance, transpiration, carbonic anhydrase and nitrate reductase activities, antioxidant enzyme activities, and proline accumulation were also significantly enhanced, suggesting improved photosynthesis, nutrient uptake, and stress tolerance. In summary, combined seed soaking and foliar application of CuO NPs substantially promoted mustard growth and physiological performance, underscoring their potential as nanofertilizers. However, further long-term field studies and environmental safety assessments are necessary before large-scale implementation.

## 1 Introduction

Nanotechnology is expected to revolutionize various research fields, with evidence supporting this. Studies range from lab experiments to applications in animal testing, agriculture, cosmetics, biomedicine, electronics, automotive, and medicine, highlighting the importance of this field (Malik et al., 2023). Nanoparticles (NPs), particles 1–100 nm in size with unique properties, are extensively studied in agriculture to boost crop productivity (Ditta et al., 2015; El-Saadony et al., 2022). As the global population grows, sustainable development and food security are vital. Nanoparticles, including nanofertilizers and nanopesticides, have been documented to promote plant growth, improve nutrient utilization efficiency, and increase tolerance to environmental stresses (Khan and Rizvi, 2017). They can be metallic or non-metallic, each with specific roles. Examining their effects on plants is important given their potential impacts on agriculture, health, and the environment (Milewska-hendel et al., 2016; Atanda et al., 2025; Santos et al., 2025). Common agricultural NPs include copper, zinc oxide, silver, aluminum, gold, silicon, cerium, and titanium, which aid nutrient delivery, reduce stress, and promote growth (Arora et al., 2012; Kaningini et al., 2022; Rossi et al., 2016; Silva et al., 2022; Thangavelu et al., 2024).

A primary concern with the use of NPs is their potential to exert both beneficial and adverse effects on plant growth and development, depending on their physicochemical properties and application methods (Altammar, 2023). While NPs can promote plant growth, protect against pathogens, and they also pose risks, including toxicity and bioaccumulation (Agrahari and Dubey, 2020). The possibility of NPs entering the food chain highlights the importance of assessing their impact on horticultural plants (Nair et al., 2010). Micronutrients delivered as nanoparticles have shown greater effectiveness in improving plant productivity and quality compared to conventional forms (Yusefi-Tanha et al., 2020). Research into various NPs, including green-synthesized and metal-based nanoparticles, is justified due to their unique characteristics, such as size, surface properties, and composition, which affect both their applications and toxicity (Imtiaz et al., 2025b; Khan et al., 2022; Shahalaei et al., 2024). Green synthesis is increasingly regarded as a sustainable, less toxic, and environmentally friendly method (Castillo-Henríquez et al., 2020) that utilizes natural plant metabolites to influence plant growth and defense mechanisms (Mawthoh et al., 2023; Tripathi et al., 2022). However, potential negative effects on plants and the environment require careful evaluation.

Copper (Cu) is essential for plant growth. CuO NPs are used in catalysts, sensors, heat transfer, semiconductors, and solar cells (Akhtar et al., 2025; Siddiqi and Husen, 2020; Singh et al., 2017). In agriculture, they serve as pesticides, herbicides, fertilizers, soil remediation agents, and growth regulators (Bakshi and Kumar, 2022; Gomes et al., 2022; Rehman et al., 2019). Among metal-based nanoparticles, copper oxide (CuO) NPs are particularly relevant because copper is an essential micronutrient involved in plastocyanin formation, lignin synthesis, and antioxidant enzyme function. Unlike non-essential metals (e.g., Ag, Cd), CuO NPs can serve dual roles as nano-nutrients and stress mitigators. Moreover, CuO NPs are more stable and cost-effective than Copper nanoparticles (Cu NPs), and their controlled release reduces the risk of phytotoxicity compared to ionic copper salts (Katarína et al., 2021). Plants may encounter CuO NPs in soil, air, or water, and these NPs may interact with roots or seeds. CuO NPs are also used in electronics, filters, ceramics, coatings, textiles, lubricants, and superconductors (Molahalli et al., 2024; Omar et al., 2024; Saleem et al., 2025). Research shows that biogenic CuO NPs can affect pigeon pea growth (Shende et al., 2017) and inhibit root growth in *Brassica nigra* seeds (Zafar et al., 2017). Studies on soybean and chickpea at up to 2000 ppm indicate that CuO NP effects vary with particle size, shape, concentration, and plant species (Adhikari et al., 2012).

Cu-based nanoparticles, such as Cu NPs and CuO NPs, are gaining attention in agriculture for their antimicrobial properties and ability to influence plant physiological and biochemical processes (López-Luna et al., 2023). Copper NPs enhance shoot dry weight, plant height, photosynthesis, and antioxidant activity across various crops and concentrations (Faraz et al., 2022; Priyanka et al., 2019; Rameen et al., 2024; Rizwan et al., 2025; Wang et al., 2023). CuNPs impact physiological, morphological, molecular, and cellular mechanisms. Recent studies demonstrate that copper oxide nanoparticles (CuO NPs) exert diverse effects on crop performance, including enhancing photosynthesis (Carlesso et al., 2025), modulating antioxidant activity (Rizwan et al., 2025), and increasing stress tolerance (López-Luna et al., 2023). Findings from key crops such as wheat, tomato, maize, and chickpea demonstrate the potential of CuO NPs to improve physiological functions and stress tolerance in challenging environmental conditions.

This study examined the combined effects of copper oxide nanoparticles (CuO NPs), administered via seed priming followed by foliar spray, on mustard growth performance. A pot experiment was conducted, and various phenotypic, physiological, and biochemical parameters were assessed.

## 2 Materials and methods

### 2.1 Seeds

Authentic seeds of *Brassica juncea* Czern & Coss cv. RGN-48 was obtained from the Indian Agricultural Research Institute, Pusa, New Delhi, India. Before each experiment, healthy and uniform-sized seeds were assessed for viability. The seeds were then surface-sterilized with a 0.01% mercuric chloride solution for 5 minutes, then rinsed at least 3 times with double-distilled water (DDW) to remove any residual mercuric chloride.

### 2.2 Preparation of a nanoparticle solution

Copper Oxide nanoparticles were obtained directly from Sigma-Aldrich Chemicals Pvt. Ltd., India, with a stated purity of 99.5% and an average particle size of <50 nanometers. The nanoparticles were dispersed in double-distilled water (DDW) through ultrasonication for thirty minutes to produce a uniform suspension prior to use. To reduce particle aggregation, small magnetic stir bars were added to the suspension to maintain continuous agitation. A stock solution with a concentration of 16 mg L^-1^ of CuO NPs was prepared, from which the required concentrations of CuO NPs were subsequently prepared for the ensuing experimental investigations.

### 2.3 Experimental design and procedure

**Figure 1:**
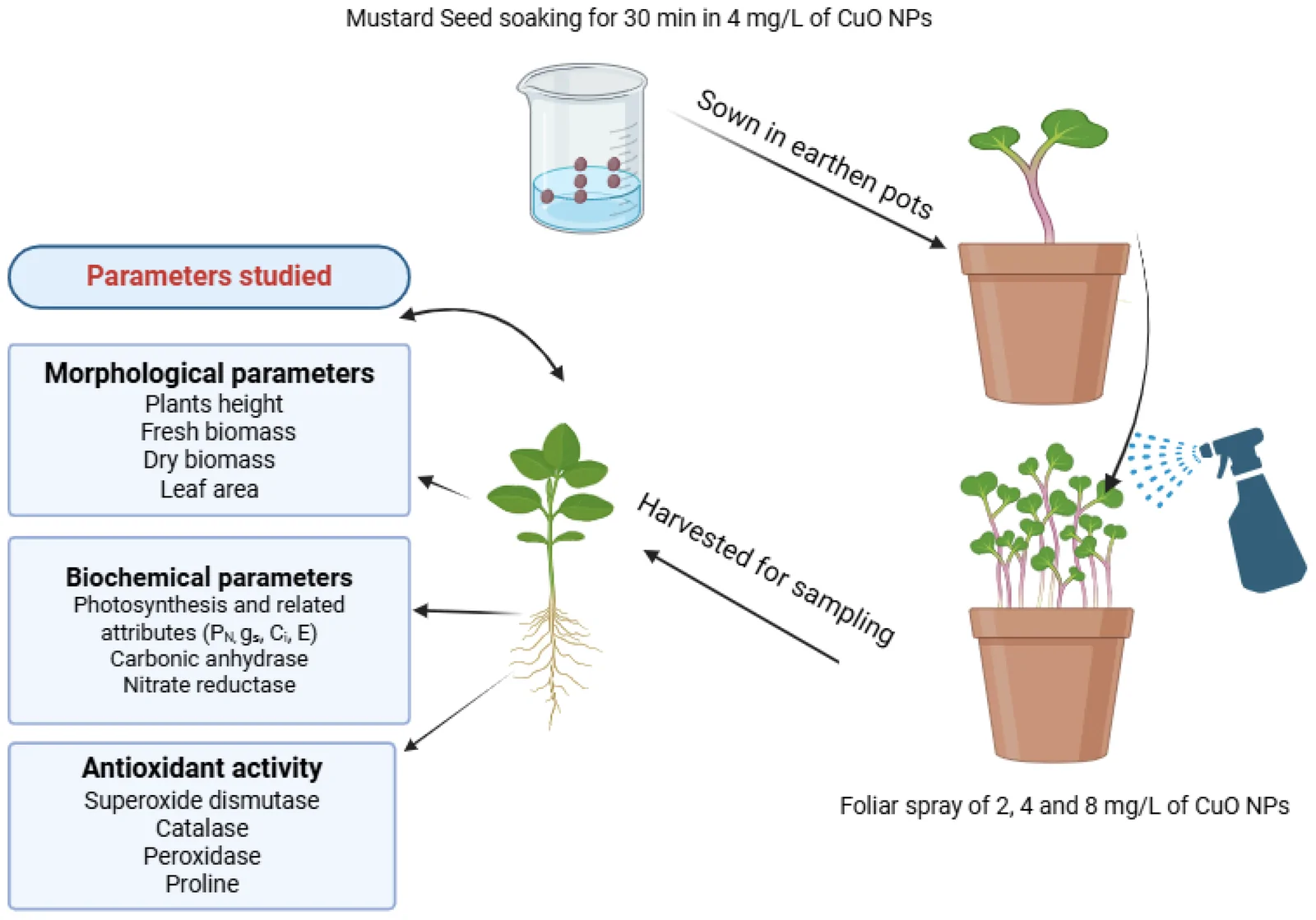
This figure presents the most basic experimental design and the parameters examined in these experiments.

Surface-sterilized seeds *of Brassica juncea* (L.) cv. RGN-48 were sown in earthen pots measuring 25 x 25 cm at a density of eight seeds per pot. The pots were filled with soil collected from the Department of Botany’s experimental field at Aligarh Muslim University. The soil was classified as sandy loam, with a pH of 7.6, electrical conductivity of 0.57 dS m^-1^, and available nutrients of 98.5 mg kg^-1^ (nitrogen), 141.1 mg kg^-1^ (phosphorus), and 7.15 mg kg^-1^ (potassium). The pots were arranged randomly in the net house. Seven days after sowing (DAS), plants were thinned to retain three uniform individuals per pot. Each treatment comprises three replicates, with three plants per pot in each replicate, which makes a total of 9 plants per treatment. Plant measurements within each pot were averaged prior to analysis. Irrigation was performed with tap water as required. The experiment was conducted during the winter season, utilizing a randomized complete block design to examine the effects of seed soaking and foliar application of CuO NPs on the growth, photosynthetic parameters, and biochemical responses of *Brassica juncea* cv. RGN-48. A simplified experimental design is illustrated in **Figure 1**.

### 2.4 Growth parameters

#### 2.4.1 Shoot and root length per plant:

Plants with intact roots were removed from each pot and immersed in a bucket of tap water. The samples were gently agitated to dislodge soil particles, and the roots were subsequently washed under running tap water. Shoot and root lengths were measured using a meter scale.

#### 2.4.2 Fresh and dry mass of shoot and root:

After washing, plants were blotted with filter paper to remove surface moisture. Roots and shoots were separated, and their fresh mass was measured using an electronic balance. Plant tissues were then oven-dried at 70 °C for 48 hours to determine dry mass.

#### 2.4.3 Leaf area

A fully expanded leaf was chosen from each plant, and the leaf area was measured with a leaf area meter (ADC Bioscientific, Hoddesdon, Herts, UK).

### 2.5 Photosynthesis and related attributes

Chlorophyll content was quantified as SPAD values using a Minolta chlorophyll meter (SPAD-502; Konica Minolta Sensing Inc., Japan) on fully expanded leaves.

The net photosynthetic rate (P*_N_*), stomatal conductance (g*_s_*), transpiration rate (E), and internal CO_2_ concentration (C*_i_*) were determined on fully expanded leaves using a portable photosynthesis system (LI-COR 6400, LI-COR, Lincoln, NE, USA). Ambient air temperature, relative humidity, CO_2_ concentration, and photosynthetic photon flux density (PPFD) were maintained at 25 °C, 85%, 600 μmol mol^-1^, and 800 μmol m^-^² s^-1^, respectively, during measurements. Data were collected between 11:00 and 12:00 hours under clear sunlight.

### 2.6 Biochemical analysis

#### 2.6.1 Leaf Nitrate Reductase (NR) activity

Nitrate reductase (NR) activity was determined using the method described by Jaworski (1971). Fresh foliage was cut into 1 cm² pieces, and 200 mg of tissue was placed into vials containing 2.5 cm³ phosphate buffer (pH 7.4), 0.5 cm³ potassium nitrate, and 2.5 cm³ of 5% isopropanol. The vials were incubated in a BOD incubator at 30 ± 2 °C in darkness for 2 hours. Subsequently, 0.4 cm³ of the reaction mixture was combined with 0.3 cm³ sulphanilamide and 0.3 cm³ NED-HCl. After 20 minutes to allow for color development, the solution was diluted to 5 cm³ with water. Absorbance was measured at 540 nm using a spectrophotometer. A blank was included in each assay. Enzyme activity was expressed as nmol NO_2_^-^ per g fresh weight (FW) per second.

#### 2.6.2 Leaf Carbonic Anhydrase (CA) activity

Carbonic anhydrase (CA) activity was measured according to the method described by Dwivedi and Randhawa (1974). Fresh leaf samples were cut into small pieces at temperatures below 25 °C. A 200 mg portion was homogenized in 10 cm³ of 0.2 M cysteine hydrochloride and maintained at 4 °C for 20 minutes. The leaf pieces were blotted dry and transferred to a test tube containing 4 mL of phosphate buffer (pH 6.8), 4 mL of 0.2 M sodium bicarbonate, and 0.2 mL of 0.002% bromothymol blue. The mixture was gently shaken and incubated at 4 °C for 20 minutes. The CO_2_ released was titrated with 0.05 N HCl using methyl red as an indicator. A blank without a leaf sample was included as a control. Enzyme activity was calculated using the standard formula.

### 2.7 Antioxidant enzyme activities

Fresh leaf tissue (500 mg) was homogenized in 5 mL of 50 mM phosphate buffer (pH 7.0) containing 1% polyvinylpyrrolidone. The homogenate was centrifuged at 15,000×g for 10 minutes at 5 °C, and the resulting supernatant was collected for enzyme assays.

#### 2.7.1 Catalase (CAT)

Catalase (CAT) activity was measured using the permanganate titration method as described by Chance and Maehly (1955). The reaction mixture contained 3 mL of phosphate buffer (pH 6.8), 1 mL of 0.1 M hydrogen peroxide, and 1 mL of enzyme extract, and was incubated at 25 °C for one minute. After incubation, 10 mL of 2% sulfuric acid was added. The remaining hydrogen peroxide was titrated with 0.1 N potassium permanganate until a stable purple color was observed for 15 seconds. A control sample was prepared by adding sulfuric acid before adding the enzyme extract.

#### 2.7.2 Peroxidase (POX) Activity

Peroxidase (POX) activity was measured following the method described by Chance and Maehly (1955). The reaction mixture consisted of 3 mL pyrogallol phosphate buffer, 0.1 mL enzyme extract, and 0.5 mL 1% H_2_O_2_. Absorbance at 420 nm was recorded at 20-second intervals over a period of 3 minutes. A control sample lacking enzyme extract was also prepared.

#### 2.7.3 Superoxide Dismutase (SOD) activity:

Superoxide dismutase (SOD) activity was measured according to the method of Beauchamp and Fridovich (1971). The 3 mL reaction mixture contained 1 mL of 50 mM phosphate buffer (pH 7.8), 0.5 mL of 13 mM methionine, 0.5 mL of 75 μM nitro blue tetrazolium (NBT), 0.5 mL of 2 μM riboflavin, 0.5 mL of 0.1 mM EDTA, and 0.1 mL of enzyme extract, with riboflavin added last. The reaction was initiated by exposing the tubes to 15-W fluorescent lamps for 10 minutes, while a non-illuminated mixture served as a blank. Absorbance was measured at 560 nm. One unit of SOD activity was defined as the amount of enzyme required to inhibit 50% of NBT photo-reduction, expressed per gram of fresh mass.

#### 2.7.4 Proline content in leaves

Proline concentration was quantified following the protocol described by Bates et al. (1973). Fresh leaf tissue (0.5 g) was homogenized in 3% sulfosalicylic acid, filtered through Whatman No. 2 paper, and the filtrate was adjusted to volume with two 5 mL washes of the same acid. 2 mL of the extract, 2 mL of glacial acetic acid, and 2 mL of acid ninhydrin were added to the extract. The mixture was heated in boiling water for 1 hour, then cooled in an ice bath. Subsequently, 4 mL of toluene was added, and the mixture was shaken for 20 to 30 seconds. The toluene layer was separated, brought to room temperature, and absorbance was measured at 520 nm using a blank as a reference.

### 2.8 Statistical analysis

Data from three independent biological replicates were analyzed using a one-way ANOVA to assess the impact of CuO NP treatments on growth, physiological, and biochemical parameters. The data were statistically analyzed, and the standard error (SE) was calculated. All statistical tests were conducted with IBM SPSS Statistics version 26. Each pot was treated as an independent experimental unit. One-way ANOVA followed by Duncan’s multiple range test (p < 0.05) was applied. Results are presented as mean ± standard error (SE).

## 3 Result

This experiment aimed to elucidate the combined effects of seed soaking and foliar spraying with CuO NPs on mustard plant performance. The summarized results are provided below.

### 3.1 Growth characteristics

**Figure 2:**
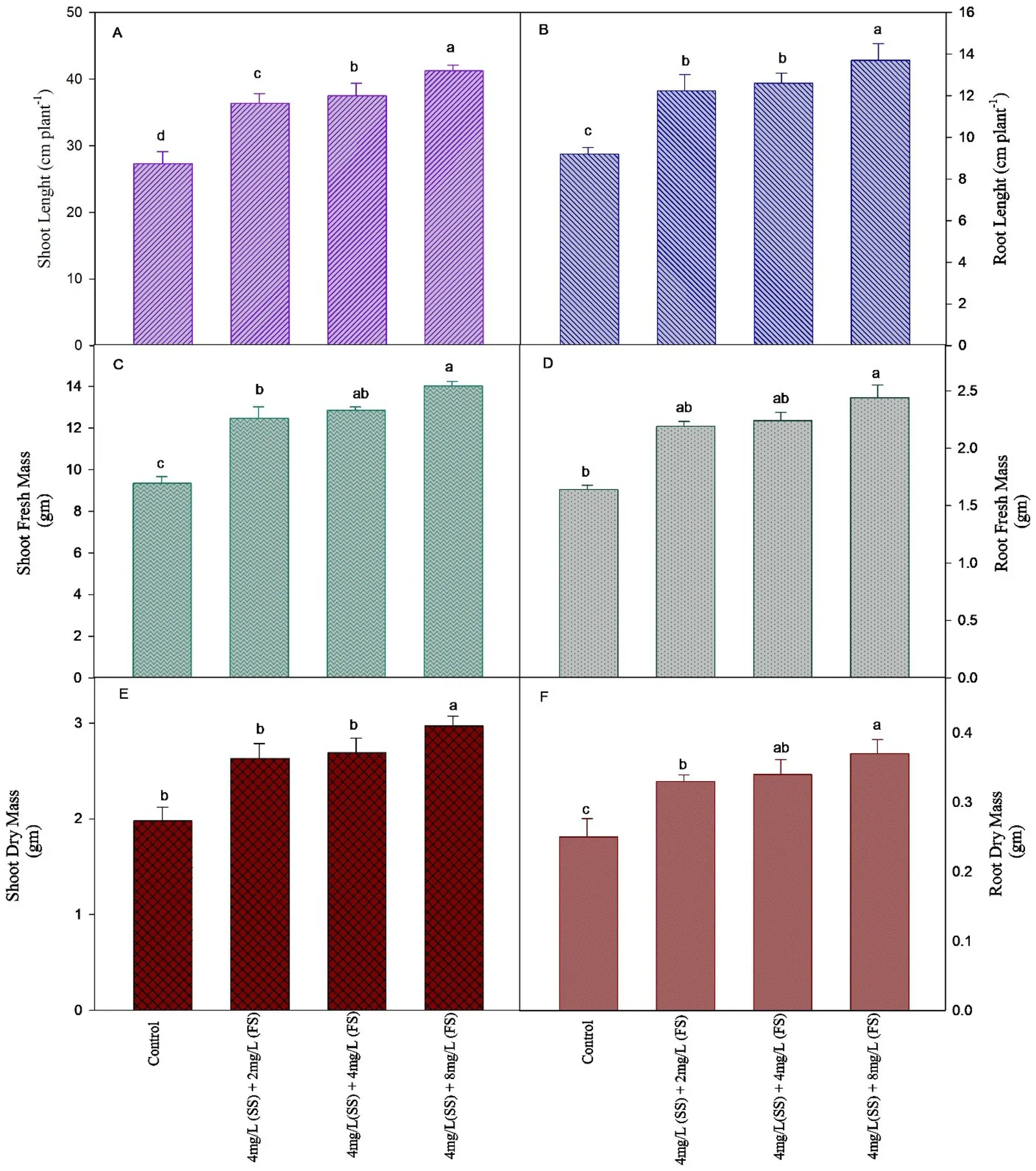
Effect of nanoparticles (NPs) on the length of shoot **(A)** and root length **(B)**, fresh mass of shoot **(C)** and root **(D)**, dry mass of shoot **(E)** and root **(F)** of mustard at 45 DAS. All data are the mean of three replicates (n = 3), and vertical bars indicate standard error (± SE). Different lowercase letters above bars indicate significant differences among treatments based on one-way ANOVA followed by Duncan’s multiple range test (*P* < 0.05).

All measured growth parameters, including root and shoot length and fresh and dry biomass, exhibited significant improvements following CuO NP treatments compared to the control (**Figures 2A–D**). The combination of seed soaking at 4 mg L^-1^ for 30 minutes and foliar spray at 8 mg L^-1^ resulted in significant enhancement. At 45 days after sowing (DAS), shoot length increased by 51%, root length by 48%, shoot fresh weight by 50%, root fresh weight by 48%, and shoot and root dry weights by 50% and 48%, respectively, relative to the control.

### 3.2 Leaf area and chlorophyll (SPAD value)

**Figure 3:**
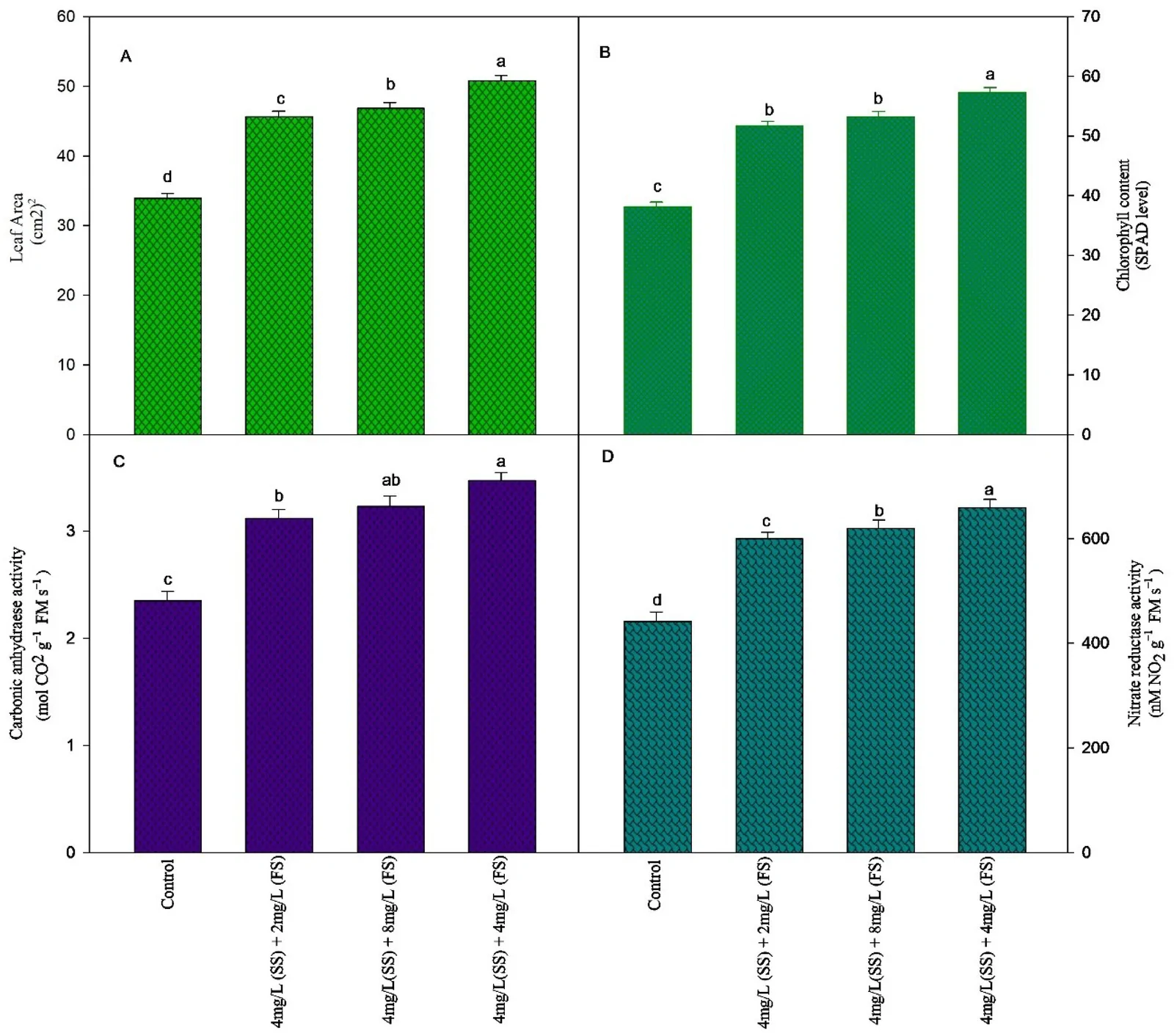
Effect of nanoparticles (nps) on the Leaf area **(A)** and SPAD Chlorophyll level **(B)**, Carbonic anhydrase activity **(C)**, and Nitrate reductase activity **(D)** of mustard at 45 DAS. All data are the mean of three replicates (n = 3), and vertical bars indicate standard error (± SE). Different lowercase letters above bars indicate significant differences among treatments based on one-way ANOVA followed by Duncan’s multiple range test (*P* < 0.05).

Leaf area and SPAD values increased significantly with both plant age and copper oxide nanoparticle (CuO NP) treatments (**Figures 3A, B**). The combined application of 4 mg L^-1^ seed soaking and 8 mg L^-1^ foliar application resulted in the highest values, demonstrating a significant improvement over the control and other treatments. At 45 days after sowing (DAS), leaf area increased by 49%, and SPAD values increased by 50% compared with the control.

### 3.3 Photosynthetic characteristics

**Figure 4:**
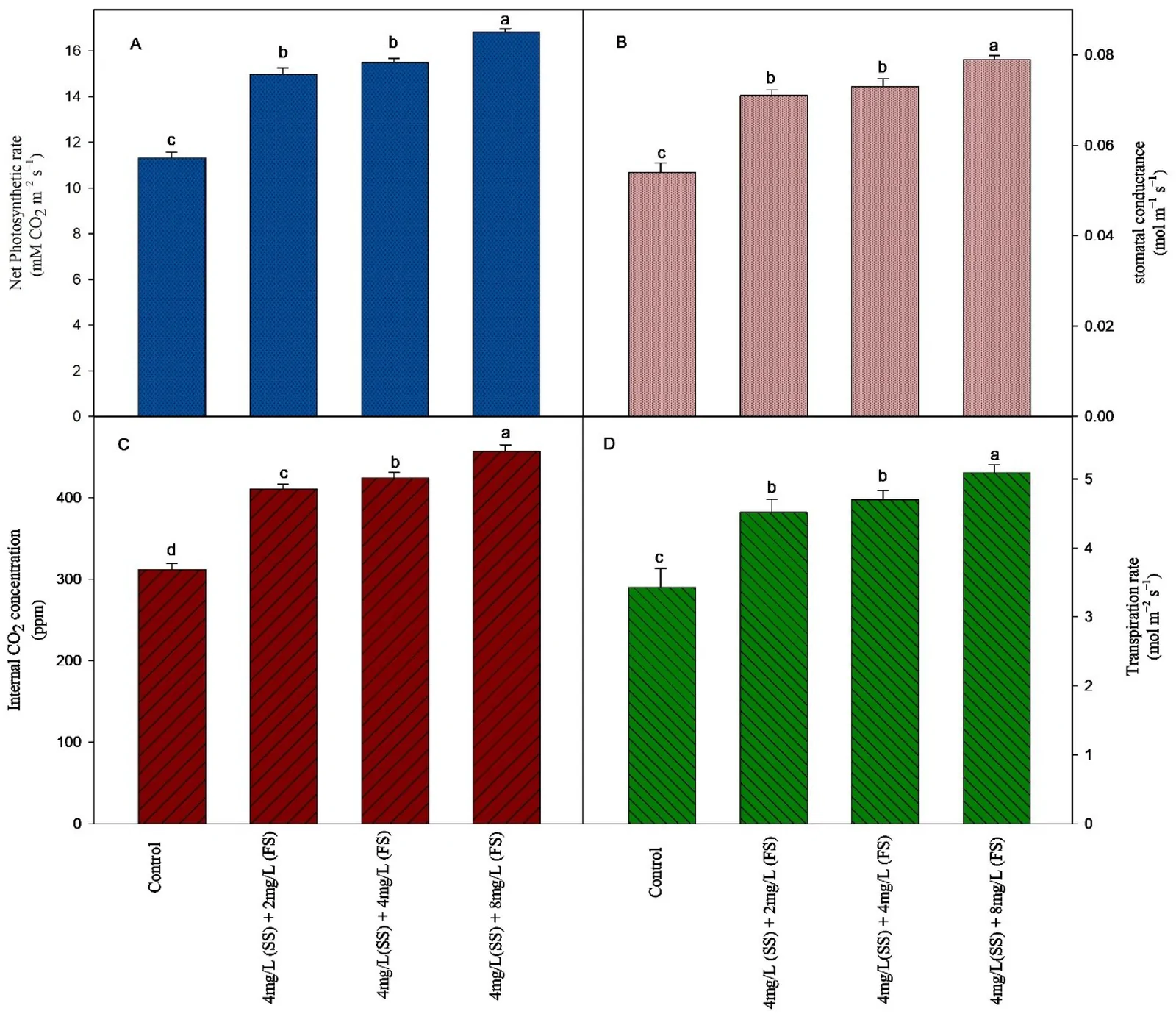
Effect of nanoparticles (nps) on the net photosynthetic rate **(A)**, stomatal conductance **(B)**, internal CO_2_ concentration **(C)**, and transpiration rate **(D)** of mustard at 45 DAS. All data are the mean of three replicates (n = 3), and vertical bars indicate standard error (± SE). Different lowercase letters above bars indicate significant differences among treatments based on one-way ANOVA followed by Duncan’s multiple range test (*P* < 0.05).

Photosynthetic parameters, including net photosynthetic rate, stomatal conductance, transpiration rate, and intercellular CO_2_ concentration, increased significantly following CuO NP treatments (**Figures 4A–D**). The greatest enhancement occurred in plants subjected to 4 mg L^-1^ seed soaking and 8 mg L^-1^ foliar spray at 45 DAS, when P*_N_*, C*_i_*, g*_s_*, and E increased by 48%, 46%, 46%, and 46%, respectively, compared to the control.

### 3.4 Activity of carbonic anhydrase (CA) and nitrate reductase (NR)

Carbonic anhydrase (CA) and nitrate reductase (NR) activities increased significantly with both plant age and copper oxide nanoparticle (CuO NP) treatments (**Figures 3C, D**). The combined treatment resulted in the highest enzyme activities. At 45 DAS, CA and NR activities increased by 47% and 49%, respectively, compared with the control.

### 3.5 Antioxidant enzymes

**Figure 5:**
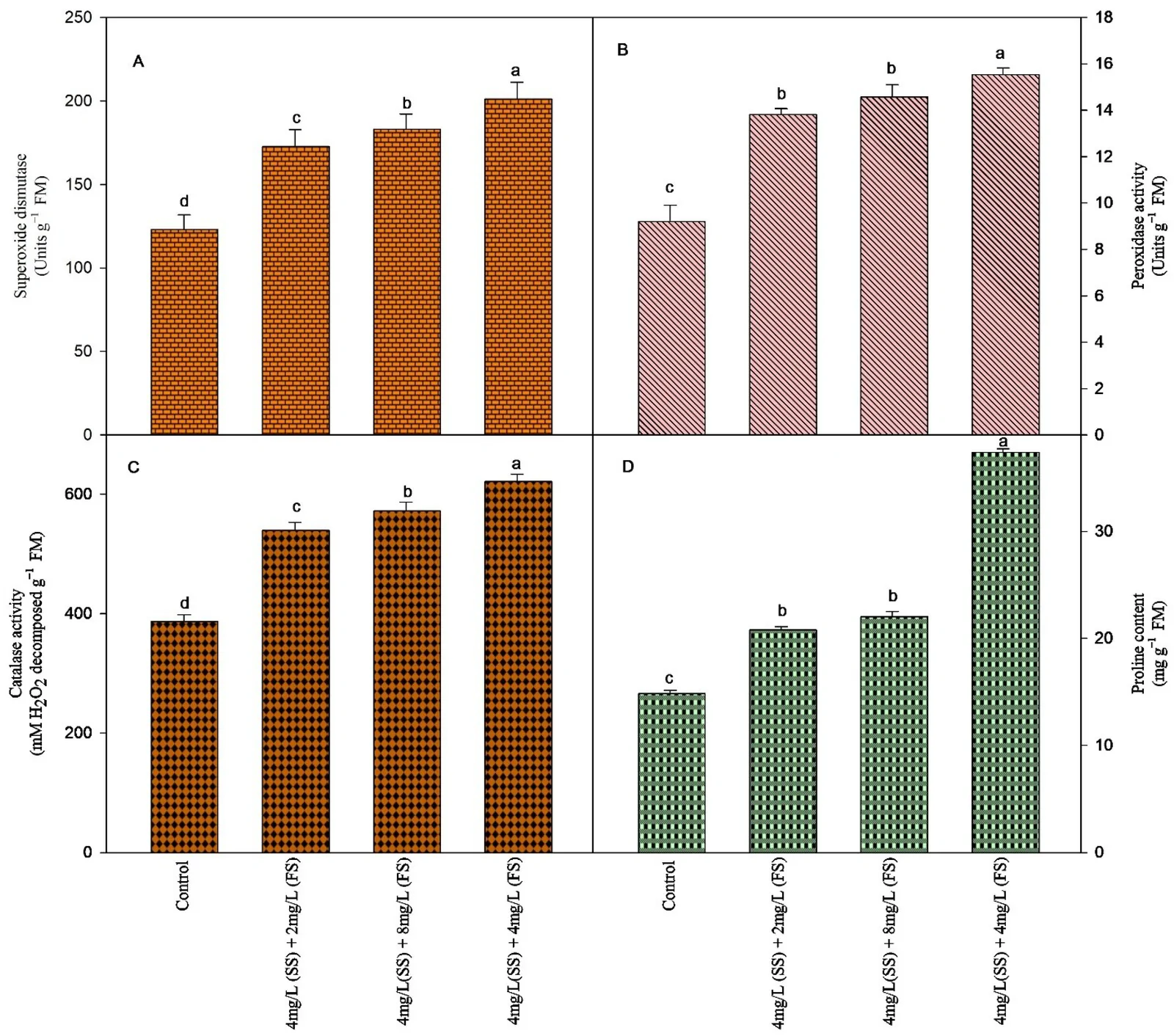
Effect of nanoparticles (NPs) on the superoxide dismutase **(A)**, peroxidase activity **(B)**, catalase activity **(C)**, and proline content **(D)** of mustard at 45 DAS. All data are the mean of three replicates (n = 3), and vertical bars indicate standard error (± SE). Different lowercase letters above bars indicate significant differences among treatments based on one-way ANOVA followed by Duncan’s multiple range test (*P* < 0.05).

The activities of catalase (CAT), peroxidase (POX), and superoxide dismutase (SOD) increased significantly in response to CuO nanoparticle (NP) treatments and advancing plant growth stages (**Figures 5A–C**). The greatest enhancement was observed with the combined application of seed soaking and foliar spray, and at 45 DAS, CAT, POX, and SOD activities increased by 60%, 68%, and 63%, respectively, compared with the control.

### 3.6 Proline content

Proline content increased significantly in response to CuO nanoparticle application and advancing plant age (**Figure 5D**). The combined treatment resulted in the highest proline accumulation. At 45 DAS, proline content was 61% higher than in the control.

## 4 Discussion

The application of copper oxide nanoparticles (CuO NPs) via seed imbibition or foliar treatment significantly enhances growth, physiological processes, and biochemical attributes in mustard plants. This improvement is attributed to the small size and large surface area of CuO NPs, which enable more efficient absorption by seeds and leaves, resulting in increased uptake, internal translocation, and bioavailability compared to larger particles (Farid et al., 2025; Murshed et al., 2014; Ulhassan et al., 2025).

Previous research has established the effects of nanoparticles on plant growth. In the present study, different concentrations and application methods of copper oxide (CuO) nanoparticles were evaluated to assess their effects on shoot length (SL), root length (RL), and biomass in mustard plants. All tested concentrations of CuO nanoparticles significantly promoted growth (**Figures 2A–F**). These results align with findings by Hafeez et al. (2015) in wheat and by (Mukherjee et al., 2014) In green peas, in which nanoparticles increased root and shoot length. The observed improvements in shoot and root length and biomass are closely associated with copper’s function as a micronutrient and enzyme cofactor in essential physiological processes. Enhanced root architecture and activation of metabolic enzymes likely facilitated increased water and nutrient uptake, carbohydrate synthesis, and overall plant development (Marschner, 2011; Vera-Reyes et al., 2018). CuO NPs are known to enter seeds via apoplastic and symplastic pathways during imbibition. Following foliar application, NPs can penetrate through stomata or cuticular cracks, translocate via phloem, and accumulate in young leaves and reproductive tissues. This explains the observed increases in leaf chlorophyll and overall vegetative growth observed following CuO NPs treatments (Avellan et al., 2021; Lv et al., 2021).

Chlorophyll content and leaf area serve as critical indicators of plant stability under stress. Chlorophyll, an essential component of photosynthesis, exhibits increased activity under favorable conditions (Taiz et al., 2015). Application of CuO nanoparticles elevated chlorophyll levels, as indicated by SPAD values, and increased leaf area across all treatments (**Figures 4A, B**). These results corroborate findings from studies involving ZnO, Fe_2_O_3_, and TiO_2_ nanoparticles (Fathi et al., 2017; Özgören Can et al., 2024). The observed increases in leaf area and chlorophyll content indicate enhanced photosynthetic capacity, likely attributable to copper’s role in enhancing plastocyanin within the photosynthetic electron transport chain. This enhancement facilitates greater electron transfer between photosystems, promotes chlorophyll biosynthesis, delays leaf senescence, and expands the photosynthetic surface area (Carlesso et al., 2025; Vera-Reyes et al., 2018).

**Figure 6:**
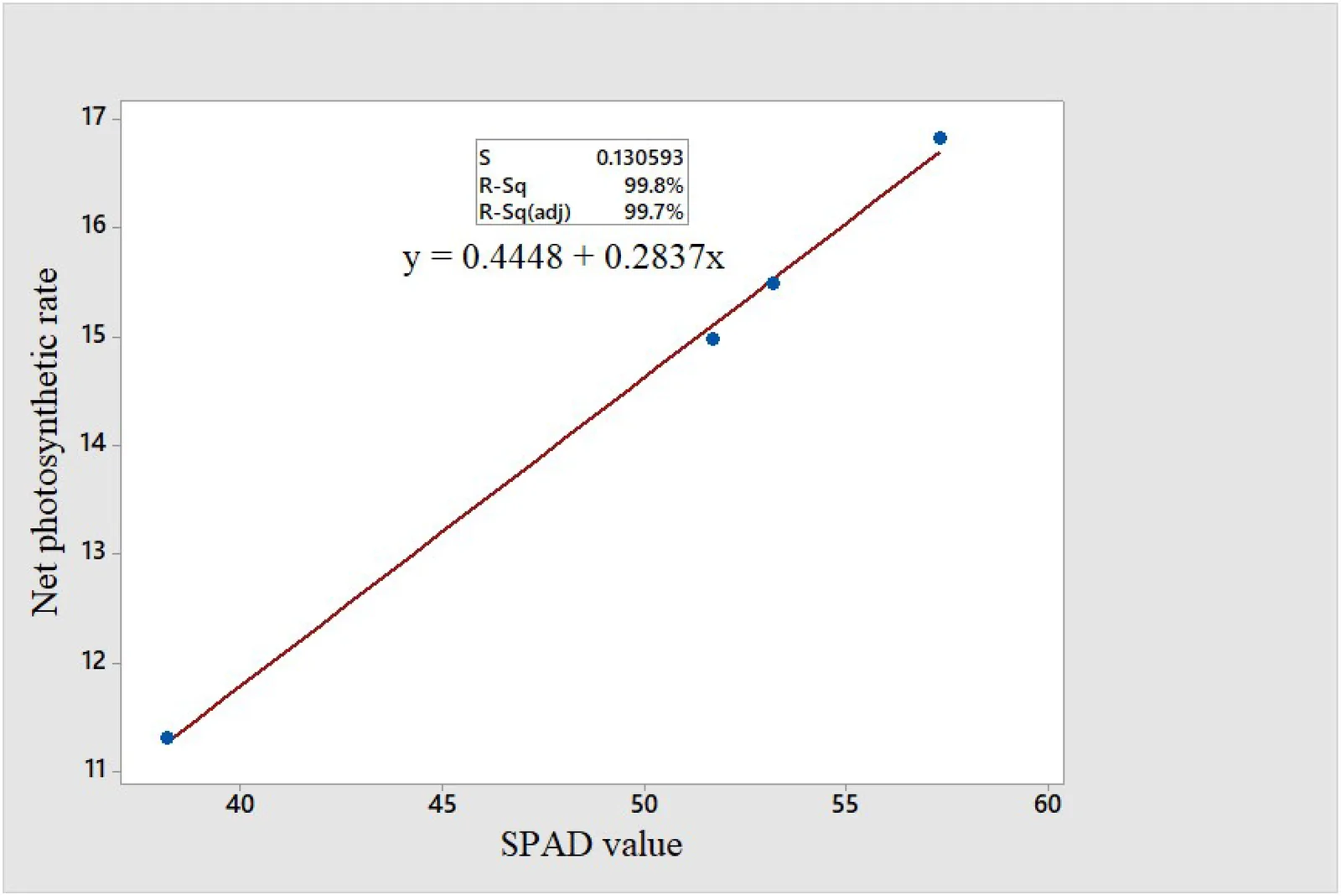
Correlation between SPAD value and net photosynthetic rate.

Photosynthesis is fundamental to plant growth. Application of CuO nanoparticles improved key physiological parameters, including P*_N_*, C*_i_*, g*_s_*, and E, indicating enhanced photosynthetic performance (**Figures 4A–D**). Increases in g*_s_* and E reflect improved stomatal regulation, which facilitates greater CO_2_ uptake and carbon assimilation. Copper-containing proteins involved in electron transport may further enhance photosynthetic efficiency. Nanoparticles may also influence guard cell signaling, thereby improving gas exchange (Burman and Kumar, 2018; Shahid et al., 2023). Enhanced photosynthetic efficiency may stem from an increase in Rubisco activity, as evidenced by (Xuming et al., 2008). They documented that nano-anatase upregulates Rubisco subunit expression, thereby augmenting Rubisco activity and nitrogen use efficiency. It is also plausible that CuO NPs contribute to this process, given reports of improved photosynthetic parameters. A strong positive correlation exists between SPAD values and P*_N_* (**Figure 6**), as higher SPAD values indicate greater chlorophyll content, thereby supporting light absorption and CO_2_ fixation. CuO nanoparticle treatments elevate chlorophyll content and light absorption, as confirmed by higher SPAD readings. These findings demonstrate that SPAD measurements can non-destructively estimate photosynthetic activity and that nanomaterials can enhance plant productivity by increasing chlorophyll content.

Carbonic anhydrase (CA), a zinc-dependent enzyme, catalyzes the conversion of CO_2_ to HCO_3_^-^ during photosynthesis and participates in ion exchange, respiration, and pH buffering (Escudero-Almanza et al., 2012). Nitrate reductase (NR) reduces nitrate to nitrite using NAD(P)H as the electron donor. Copper oxide nanoparticles (CuO NPs) enhance the activities of both CA and NR, thereby improving carbon and nitrogen metabolism (**Figures 3C, D**). Similar enhancements have been observed with SiO_2_, TiO_2_, and ZnO nanoparticles, which increase CA activity in tomatoes, Cucurbita pepo, and peppermint (Farid et al., 2025; Imtiaz et al., 2025a). Nanoparticles also increase NR activity, likely by inducing gene expression, as demonstrated with silver nanoparticles in beans (Prażak et al., 2020). CA maintains a steady supply of CO_2_ for photosynthesis, while NR is essential for nitrate assimilation. Increased enzyme activity reflects improved metabolism, including increased acid production, protein production, and copper-mediated activation or stabilization, leading to greater amino acid and protein synthesis and supporting plant growth.

Reactive oxygen species (ROS) are byproducts of oxygen metabolism in plants and play essential roles in maintaining homeostasis and facilitating signaling processes (Mansoor et al., 2022). The reduction of O_2_ generates ROS, including superoxide (O_2_^-^), singlet oxygen (¹O_2_), hydroxyl radicals (HO), and hydrogen peroxide (H_2_O_2_) (Krystynik, 2021). Mustard seeds treated with 4 mg L^-1^ and sprayed with 8 mg L^-1^ CuO nanoparticles exhibited increased activities of superoxide dismutase (SOD), peroxidase (POX), and catalase (CAT) (**Figures 5A–C**), likely resulting from nanoparticle uptake and subsequent changes in nutrient availability, gene activation, or oxidative processes (Hossain et al., 2015). These findings are consistent with evidence that nanoparticles stimulate the biosynthesis of antioxidant enzymes across various nanoparticle types (Gupta et al., 2018; Jomova et al., 2024; Zhang et al., 2019). Enhanced SOD activity facilitates O_2_ radical scavenging, while increased CAT and POX activities strengthen antioxidant defenses, neutralize ROS, protect cellular structures, and maintain redox balance (Dimkpa et al., 2012; Gill and Tuteja, 2010). Recent studies have demonstrated that copper oxide nanoparticles (CuO NPs) can upregulate genes encoding superoxide dismutase (SOD), catalase (CAT), and ascorbate peroxidase (APX), which correlates with our observed increases of 60–68% in enzyme activities. Moreover, proline accumulation induced by nanoparticles (61%) is now recognized as an osmolyte that supports signaling functions, rather than merely a passive marker of stress (Kaleem et al., 2025).

Proline contributes to plant stress management by protecting cellular membranes and enzymes, scavenging free radicals, chelating metals, and mediating signaling responses (Ghosh et al., 2022; Zulfiqar and Ashraf, 2023). In this study, mustard plants treated with CuO nanoparticles exhibited a significant increase in proline levels, indicating activation of stress responses and enhanced tolerance, a pattern commonly observed under nanoparticle-induced stress (**Figure 5D**). The observed increase in proline is considered an adaptive and protective response, rather than an indicator of toxicity, as it was accompanied by enhanced antioxidant enzyme activity, improved growth, and increased photosynthetic efficiency. These results indicate that CuO NPs at the tested concentrations induced mild, beneficial hormetic stress, thereby enhancing stress resilience. This observation is consistent with previous research demonstrating elevated proline levels in various crops exposed to nanoparticles such as SiO_2_, ZnO, and TiO_2_ (Cevik, 2023; Mustafa et al., 2022; Parveen and Siddiqui, 2022; Siddiqui et al., 2019). These findings suggest that nanoparticles promote proline accumulation, thereby mitigating stress. Additionally, TiO_2_ nanoparticles in barley (Karami and Sepehri, 2018) and ZnO nanoparticles in tomato (Faizan et al., 2020) have been shown to increase proline levels, supporting osmotic adjustment, metabolic activity, and improved plant resilience under CuO nanoparticle exposure. Copper oxide nanoparticles (CuO NPs) regulate proline accumulation and osmotic adjustment through several interconnected molecular and biochemical mechanisms. The dissolution of Cu^2+^ ions and the generation of reactive oxygen species (ROS) by these nanoparticles activate mitogen-activated protein kinase (MAPK) cascades and calcium-dependent signaling pathways. These signaling events upregulate the expression of Δ¹-pyrroline-5-carboxylate synthetase (P5CS), the rate-limiting enzyme in glutamate-derived proline biosynthesis (Sharma et al., 2019). Furthermore, CuO NPs inhibit proline dehydrogenase (*ProDH*) activity, thereby reducing proline catabolism and increasing net proline accumulation (Hayat et al., 2012). Overall, nanoparticles appear to protect plants by elevating levels of compatible solutes such as proline.

**Figure 7:**
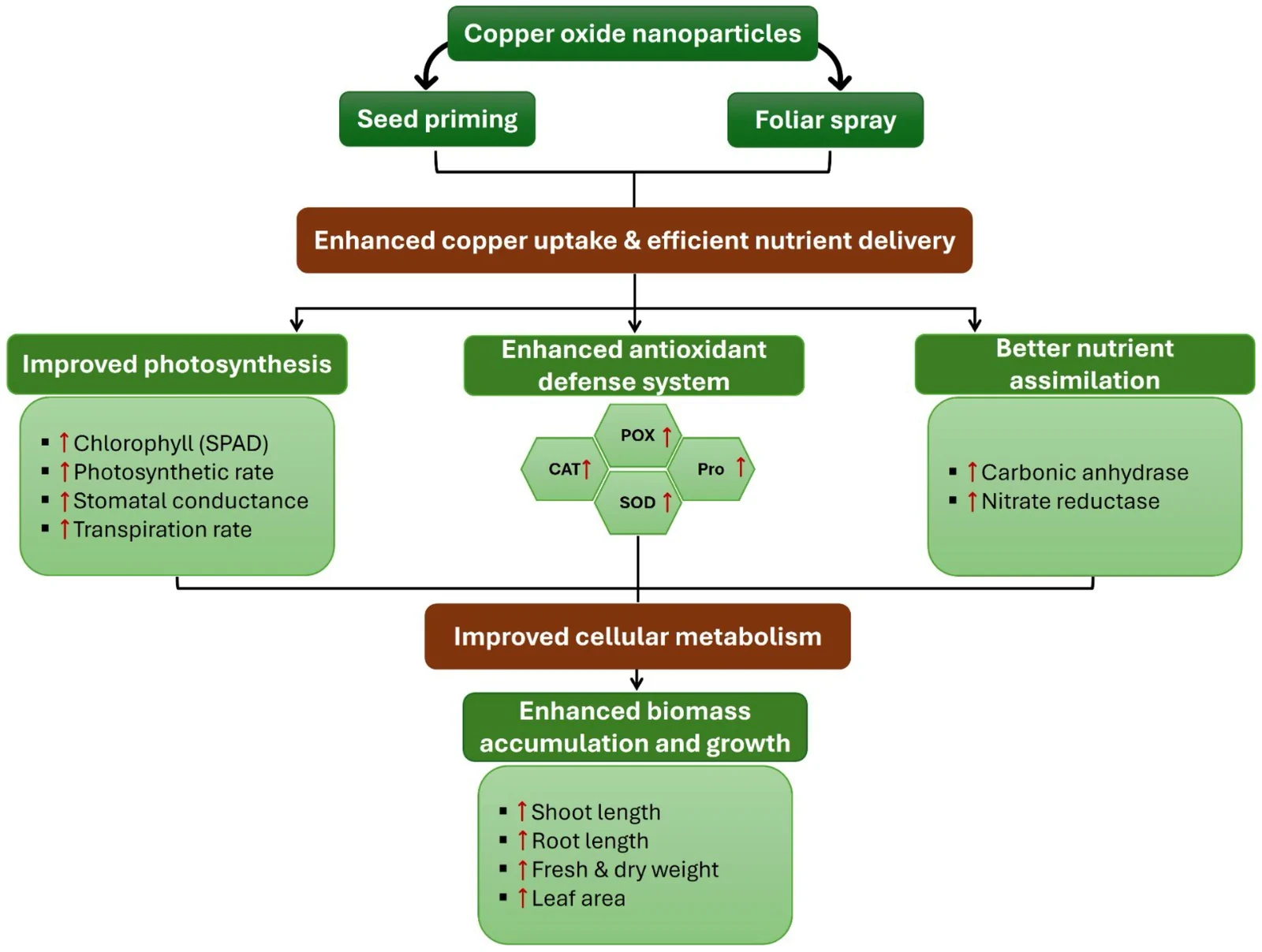
This diagram provides a comprehensive summary and a possible mechanism of CuO NPs, highlighting their role in enhancing plant growth and photosynthesis and in providing protection against oxidative stress. (CAT, catalase; POX, peroxidase; SOD, superoxide dismutase; Pro, Proline).

The results indicate that CuO nanoparticles enhance plant health through multiple mechanisms, including improved nutrient uptake, enhanced photosynthetic activity, and increased carbon and nitrogen assimilation. The findings show that CuO nanoparticles improve plant health through several mechanisms. **Figure 7** presents a schematic overview of how CuO nanoparticles enhance plant growth and physiological performance. They also help regulate stomatal function and strengthen antioxidant defenses. Combined seed and foliar applications appear particularly effective in maximizing these benefits. CuO NPs are known for their antifungal and micronutrient benefits; however, their release and buildup in soils raise serious concerns about soil microorganisms, plant health, and trophic transfer. Chronic exposure to CuO NPs has been shown to inhibit beneficial microbial enzymes and decrease the abundance of nitrogen-cycling bacteria, thereby threatening soil fertility (Rajput et al., 2021). The dissolution of Cu^2+^ ions and the inherent toxicity of particles can result in bioaccumulation in crops and animals throughout the food chain. In the rhizosphere, processes such as sulfidation or the formation of organic coatings may modify nanoparticle bioavailability, yet the effects of these transformations under field conditions remain insufficiently understood. The lack of extensive long-term studies and risk evaluations indicates that using CuO NPs in agriculture could cause unforeseen ecological effects. This underscores the importance of adopting a cautious approach. Additional research is needed to evaluate their long-term environmental impacts and safety, ensuring they provide overall benefits.

This study shows that CuO nanoparticles benefit vegetative growth, photosynthesis, nutrient metabolism, and antioxidant defenses at 45 DAS. However, their impact on reproductive development and final yields remains unassessed. Further comprehensive research, covering entire growth cycles, multiple trials, and field validation, is essential before recommending CuO nanoparticles for improving crop productivity.

## 5 Conclusion

The application of copper oxide nanoparticles (CuO NPs) via seed soaking and foliar spray significantly enhances the growth, physiological functions, and biochemical parameters of mustard plants. The most effective treatment, involving a concentration of 4 mg L^-1^ for seed soaking for 30 minutes combined with 8 mg L^-1^ of CuO NPs for foliar spray, results in increased biomass, leaf area, and chlorophyll content. It also improves photosynthetic activity and elevates the enzymatic activities of carbonic anhydrase and nitrate reductase, indicating enhanced carbon and nitrogen metabolism. Furthermore, this treatment reinforces antioxidant systems, specifically catalase (CAT), peroxidase (POX), and superoxide dismutase (SOD), and increases proline accumulation, thereby improving stress resilience and physiological stability. CuO NPs serve as effective nanomicro-nutrient sources by increasing nutrient availability, enhancing photosynthetic efficiency and enzyme activity, and providing protection against oxidative stress. The integrated methodology of seed priming and foliar application demonstrates notable effectiveness; however, before recommending it to farmers, additional research is needed to assess its long-term safety and practical feasibility under field conditions.

This study offers a preliminary evaluation of the effects of the tested treatment under defined experimental conditions. The findings should be interpreted in light of several limitations, including the lack of independent experimental replications and a small sample size. Consequently, these results are considered provisional and require validation through future research involving independent replications, larger sample sizes, and assessments across diverse environmental and field conditions. Such investigations are essential to establish the reproducibility, robustness, and generalizability of the observed responses. Furthermore, comprehensive long-term evaluations of CuO nanoparticle treatments are necessary to determine their effects on crop yield, agronomic performance, and the sustainability of agricultural production.

## Data Availability

The raw data supporting the conclusions of this article will be made available by the authors, without undue reservation.
